# Fusaric acid induced cell death and changes in oxidative metabolism of *Solanum lycopersicum* L

**DOI:** 10.1186/s40529-014-0066-2

**Published:** 2014-08-27

**Authors:** Vivek Kumar Singh, Ram Sanmukh Upadhyay

**Affiliations:** Laboratory of Mycopathology and Microbial Technology, Centre of Advanced Study in Botany, Banaras Hindu University, Varanasi, 221005 India

**Keywords:** Cell death, Fusaric acid, Hypersensitive response, Reactive oxygen species

## Abstract

**Background:**

Fusaric acid (FA) has been shown to stimulate the rapid development of disease symptoms, such as necrosis and foliar desiccation. In this study, we have evaluated the phytotoxicity of FA on tomato plants (*Solanum lycopersicum* L.). FA induced necrotic lesions in detached leaves, which are reminiscent of hypersensitive response (HR) lesions induced by plant-pathogen interactions and other abiotic stress factors.

**Results:**

FA-treated tomato leaves exhibited visible necrotic lesion as a result of cell death which was evident by Evans blue staining, enhanced reactive oxygen species (ROS) levels and DNA degradation. Changes in the generation of O_2_^.-^ and H_2_O_2_ as well as the activities of superoxide dismutase (SOD), catalase (CAT) and ascorbate peroxidase (APX) were examined in FA-treated tomato leaves. It was observed that FA exposure stimulated oxidative burst in the leaves, resulting in a lasting activation of O_2_^.-^ and H_2_O_2_ production. After first day of FA application, the H_2_O_2_ scavenging enzymes CAT and APX showed a strong activity decrease followed by gradual recovery to the control level after 2 and 3 days.

**Conclusion:**

A concomitant increase in ROS production, the down regulation of antioxidative enzymes activities and upregulation of lipid peroxidation were crucial for the onset of cell death. These results suggested that FA-induced damage might result from ROS pathways. Thus, our experiments provide a useful model plant system for research on FA-induced plant cell death.

**Electronic supplementary material:**

The online version of this article (doi:10.1186/s40529-014-0066-2) contains supplementary material, which is available to authorized users.

## Background

Fusaric acid (FA), also known as 5-butylpicolinic acid, is a non-host specific phytotoxin of *Fusarium* species and suspected of being involved in pathogenecity (Gäumann [1957], Gäumann [1958]). FA has been shown to stimulate the rapid development of some disease symptoms, such as interveinal necrosis and foliar desiccation (Sutherland and Pegg [1992]). It has been detected in plants after *Fusarium* attack and was present in much higher concentration in plant tissues infected with a virulent strain than in those infected with an avirulent one (Harborne [1989]). At the subcellular level, FA has been found to induce many physico-chemical effects (Kuzniak et al. [1999]; Wu et al. [2008]; Sapko et al. [2011]). The effects of FA have also been described in terms of changes in the proton electrochemical gradient across the plasma membrane, increase in loss of electrolytes, decrease in cellular ATP level, and inhibition of some metalloenzymes (e.g. cytochrome oxidase) leading to respiratory impairment (Marre et al. [1993]; D’Alton and Etherton [1984]; Arias [1985]). Most studies have reported that FA shows toxic effects at concentration greater than 10^-5^ M. FA participates in fungal pathogenicity by decreasing plant cell viability. However, FA is also produced by non-pathogenic *Fusaria*, potential biocontrol agents for vascular wilt diseases in which FA at non-toxic concentrations (below 10^-6^ M) activates signal transduction components necessary for plant defense responses that contribute to biocontrol activity (Bouizgarne et al. [2006]).

Plants have developed complex protection systems to cope with pathogen attack. It has been reported that reactive oxygen species (ROS) and the enzymatic systems that govern their metabolism, for example superoxide dismutase (SOD), catalase (CAT) and peroxidases may play a role in pathogenesis (Baker and Orlandi [1995]). ROS, which are rapidly produced in plant cells after pathogen attack, are potentially involved in many defense processes including the hypersensitive response (HR), phytoalexin synthesis and oxidative cross-linking of plant cell wall proteins. Many non-host selective microbial phytotoxins are known to induce a generation of ROS and in many cases, disease symptom development can be explained by this mechanism (Heiser et al. [1998]). Therefore, the possibility that ROS might also be involved in the response to FA has been of interest.

Several lines of evidences suggest that, from among the generated ROS, H_2_O_2_ plays a central role in these plant defense responses (Mehdy et al. [1996]). On the other hand ROS exhibit deleterious impact on the cellular components and so plants possess effective mechanisms for detoxification of ROS in order to protect themselves from the toxic effects. SOD catalyze the dismutation of O_2_^.-^ radicals to O_2_ and H_2_O_2_, and CAT and ascorbate peroxidase (APX) remove H_2_O_2_, thus playing a role of antioxidative key enzymes. Moreover, the Guaiacol type peroxidases may use H_2_O_2_ in the process of cell wall reinforcement by catalyzing the polymerization of cinnamyl groups into lignin, suberization and deposition of phenolic compounds. Hence the balance between ROS generation and breakdown may be critical for the outcome of plant-pathogen interaction. After failure of this balance, ROS accumulation occurs. Recently, the prominent role of ROS has been revealed in the induction, signalling and execution of plant cell death (Jabs [1999]; Apel and Hirt [2004]; Karuppanapandian et al. [2011]). The initial lower level of ROS may stimulate the production of higher amount of ROS through feedback regulation. The change of ROS content can trigger different signalling cascades leading to cell death (Breusegem and Dat [2006]). Moreover, there was a strong interplay between ROS and other signalling molecules (phytohormones) such as ethylene and salicylic acid (SA) during cell death (Overmyer et al. [2005]). Toxic level of ROS usually react with numerous cell components, causing a cascade of oxidative reactions and the consequent inactivation of enzymes, protein degradation and nucleic acid damage. In addition, excessive ROS can react with membrane lipids, generating lipid peroxides that can in turn lead to further damaging intermediates, such as malondialdehyde (MDA), to the point of inducing cellular dysfunction and/or cell death (Paciolla et al. [2004]; Maksymiec and Krupa [2006]; Schraudner et al. [1998]).

The present investigation portrays the phytotoxic action of FA towards tomato (*Solanum lycopersicum* L.). HR-like lesions were observed in excised leaves after FA infiltration. As the first step in elucidating the possible mechanisms of toxicity of FA against tomato, we analyzed the role of ROS in response to FA and the changes in the levels of several antioxidant enzymes triggered by FA stress. Microscopy and staining techniques were used to verify the toxicity and to identify the visualization of H_2_O_2_ and O_2_^.-^*in situ*. Our experiments provide a useful model system for the research on plant cell death resulting from FA.

## Methods

### Plant material and growth conditions

Tomato (*Solanum lycopersicum* L.) belongs to Solanaceae family and is an important model plant. The seeds of tomato (cv. Sel-7, susceptible to *Fusarium oxysporum* f. sp. *Lycopersici*) were obtained from Indian Institute of Vegetable Research, Varanasi, India and plants were grown in soil in green house with 14 h light and 10 h dark regime, at 27 ± 0.5°C. The plants were grown in thermocol pots of 6 cm diameter and 15 cm height containing autoclaved soil. The plants were allowed to grow and when they attained 6-8 leaves (3-4 weeks old), they were treated with FA.

### FA treatment

Fusaric acid was extracted and purified from the culture filtrate of *Fusarium oxysporum* f. sp. *lycopersici* (causal agent of Fusarium wilt of tomato) (Venter and Steyn [1998]) grown at 25°C in 500 ml flasks containing 100 ml potato dextrose broth (PDB) and then analysed by TLC (Stefan [2005]) and HPLC (Venter and Steyn [1998]). The purified and characterized FA was stored at 4°C until further use. Crystalline FA standard (5-butylpicolinic acid) was supplied by MP Biomedicals, CA, USA.

Fusaric acid treatment of tomato leaves was performed by infiltration of leaf tissue with the help of a 1 ml syringe fitted with 25 guage needle in the midrib just above the petiole on the lower side of the leaf. One ml FA solution was infiltrated to each leaflet of fully expanded tomato leaves (Kuzniak et al. [1999]). Control plants were infiltrated with sterile distilled water. The second and third fully expanded leaves from the plant base were used for the experiments. Oxidative burst, antioxidant enzyme activities, lipid peroxidation and ethylene evolution in the leaves of tomato plants were measured at 0, 4, 8, 12, 16, 20, 24, 36, 48 and 72 h after FA treatment.

### Measurement of cell death

Evans blue staining was used as a marker of cell death and cell death was measured in terms of Evans blue uptake 72 h after infiltration with various concentrations of FA (50 μg/ml, 100 μg/ml, 150 μg/ml, 200 μg/ml, 250 μg/ml and 300 μg/ml). Control leaves were infiltrated with sterile distilled water.

Evans blue staining to assess cell death was carried out by gentle heating the FA-treated and control leaves for 1 min in a freshly prepared solution of phenol, lactic acid, glycerol and distilled water (1:1:1:1) containing 20 mg/ml Evans blue. After boiling, leaf tissues were then clarified overnight in a solution of 2.5 gm/l chloral hydrate on a platform shaker at 27°C and 160 rpm. The leaf tissues were then transferred on glass slides and observed under the light microscope to see the amount of cell death in FA-treated as well as control leaves.

To estimate the amount of cell death in tomato leaves induced by FA treatment, the methodology of Baker and Mock ([1994]) was followed. The FA-treated and control leaf tissues were submerged in 10 ml plastic beakers containing 1 ml of 0.25% Evans blue and incubated on a platform shaker at 27°C and 80 rpm for 20 min. The beaker contents were poured into a small Buchner funnel and the tissues were rinsed well with deionized water until no more blue stain was eluted. The tissues were then transferred to 1.5 ml microfuge tubes. One half ml of 1% aqueous sodium dodecyl sulphate (SDS) was added in each tube to release the trapped Evans blue from the cells. The tissues were then ground using a pestle-mortar and the homogenates were diluted with 0.5 ml deionized water. The tubes were vortexed and centrifuged at 9000 *g* for 3 min. 0.8 ml aliquot of the supernatant was removed and optical density was determined spectrophotometrically (UV-VIS Spectrophotometer 2202, Systronics, India) at 600 nm.

### Nitro blue tetrazolium reducing activity

Detached leaves from the plants were subjected to the treatments and their respective controls were immersed in 50 mM potassium phosphate buffer (pH 7.8) containing 0.1% nitroblue tetrazolium (NBT) and 10 mM sodium azide (NaN_3_). The leaves were vacuum infiltrated during 2 min and then incubated for 2 h in the dark (without vacuum) and further immersed in 96% (v/v) ethanol to completely eliminate the chlorophyll. Superoxide production was visualized as a purple formazan deposit within the leaf tissues. The leaves of the plants that received no treatment were also infiltrated with 50 mM potassium phosphate buffer (pH 7.8) containing only 10 mM NaN_3_ that served as control.

Measurement of NBT reduction, a method used for the determination of O_2_^.-^, was as described by Doke ([1983]). FA-treated as well as control leaves were immersed in 3 ml 10 mM potassium phosphate buffer (pH 7.8) containing 0.05% NBT and 10 mM NaN_3_ for 1 h. Then the mixture was heated at 85°C for 15 min. and cooled rapidly. The activity of leaf tissues to reduce NBT was expressed as increased absorbance at 580 nm per h per g fresh weight (FW).

### Estimation of H2O2 level

Leaves treated with FA were cut with razor blade 1 cm above the base of the petiole and immediately placed in a beaker containing 1 mg/ml 3,3′-diaminobenzidine hydrochloric acid (DAB-HCl), adjusted to pH 5.6 with NaOH and were incubated in a humid growth chamber for 8 hours in the dark. The leaves were kept in the vertical position with 5 mm of the basal part dipped into the DAB solution. After DAB uptake, the leaves were cleared in 96% boiling ethanol, and examined with light microscope. H_2_O_2_ is visualized as a reddish-brown coloration.

The H_2_O_2_ produced was determined according to Sagisaka ([1976]). The FA-treated and control plant leaf tissue were homogenized in 5% cold trichloro acetic acid (TCA) and the homogenate was centrifuged at 17000 *g* for 10 min at 0°C. The reaction mixture contained 1.6 ml of supernatant, 0.4 ml of 50% TCA, 0.4 ml of ferrous ammonium sulphate and 0.2 ml of potassium thiocynate. The absorbance was recorded at 480 nm after 15 min of incubation. The amount of H_2_O_2_ was estimated by a calibration curve prepared with known concentrations of H_2_O_2_.

### Determination of lipid peroxidation

Lipid peroxidation was determined by measuring the amount of MDA (Heath and Packer [1968]), the end product of peroxidation of polyunsaturated fatty acids after treatment with various cell death inducers. The FA-treated and control leaf tissues were cut into small pieces and homogenized by the addition of 1 ml of 0.1% cold TCA. The homogenates were then transferred into fresh tubes and centrifuged at 10000 g for 20 min at room temperature. To 1 ml supernatant, 1 ml 20% TCA containing 0.5% thiobarbituric acid (TBA) (freshly prepared) and 0.01 ml butylated hydroxyl toluene (BHT) prepared 4% solution in ethanol were added into a new tube and incubated at 96°C for 35 min. The tubes were then transferred into ice bath and kept for 5 min followed by centrifugation at 10000 *g* for 5 min. The absorbance of the supernatant was recorded at 532 nm and corrected for non-specific turbidity by substracting the absorbance at 600 nm. The concentration of MDA was calculated from its molar extinction coefficient 156 mM/cm.

### Ethylene production assay

For ethylene production, the FA-treated and control leaf tissues of tomato plants were excised and placed in test tubes for 30 min to allow the escape of wound ethylene (Lund et al. [1998]). After this the tubes were sealed for 2 h. Gaseous samples were then analysed for ethylene content on gas chromatograph (Model CP-3800 GC, Varian, Inc. CA, USA) equipped with an flame ionization detector (FID) and a column (CP-Pora PLOT Q, 25 m × 0.32 mm) packed with fused silica. For measuring ethylene content 1 ml gas sample was injected into the GC column using a Hamilton gastight syringe (Model: 701 RN). The column, injector and detector temperatures were set at 110°C, 130°C and 130°C respectively. Nitrogen gas at a flow rate of 30 ml/min was used as the carrier. The ethylene content was quantified by comparison of peak areas with standard curve constructed from known amounts of ethylene gas (SSG, Alltech Asso. Inc. USA).

### Assays of antioxidative enzyme activities

The leaves were homogenized (1:5 w/v) in 1 M NaCl in 50 mM potassium phosphate buffer (pH 7.0) containing 1% polyvinyl pyrrolidone (PVP) and 1 mM EDTA. For assay of APX, leaf extracts were prepared in the same medium containing 1 mM sodium ascorbate. The homogenate was centrifuged and the supernatant was used as an enzyme extract to assay SOD, CAT and APX activities.

SOD activity assay was based on the method of Beauchamp and Fridovich ([1971]) which measures the inhibition of the photochemical reduction of NBT. In the spectrophotometric assay, the 3 ml mixture contained 50 mM potassium phosphate buffer (pH 7.8), 13 mM L-methionine, 75 μM NBT, 0.1 mM EDTA, 2 μM riboflavin and enzyme extract. The riboflavin was added last and the reaction was started by placing the tubes under two 15 W fluorescent lamps. The reaction was terminated after 10 min by removal from the light source. Non-illuminated and illuminated tubes without enzyme extract served as control. The absorbance was measured at 560 nm. The volume of enzyme extract corresponding to 50% inhibition of the reaction was considered as one unit of enzyme activity.

CAT activity was measured spectrophotometrically according to Dhindsa et al. ([1981]). The assay mixture contained 50 mM potassium phosphate buffer (pH 7.0), 15 mM H_2_O_2_ and enzyme extract. The decomposition of H_2_O_2_ (absorbance coefficient of 45.2/mM cm) was measured at 240 nm at 25°C.

APX was assayed following the oxidation of ascorbate to dehydroascorbate at 265 nm (an absorbance coefficient of 13.7/mM cm) by a modified method of Nakano and Asada ([1981]). The assay mixture contained 50 mM potassium phosphate buffer (pH 7.0), 0.25 mM sodium ascorbate, 25 μM H_2_O_2_ and enzyme extract. Addition of H_2_O_2_ started the reaction. The rates were corrected for the non-enzymatic oxidation of ascorbate by the inclusion of reaction mixture without enzyme extract.

Protein was determined by the method of Bradford ([1976]), with standard curves prepared using bovine serum albumin (Sigma).

### Plant genomic DNA extraction and analysis

Genomic DNA from the FA-treated and control plant leaf tissue was isolated by Doyle and Doyle CTAB method (Doyle and Doyle [1987]). For DNA extraction, 7.5 ml of CTAB isolation buffer was preheated in a 30 ml centrifuge tube to 60°C in a water bath. 1.0 g leaf tissue was ground in a 60°C CTAB isolation buffer in a preheated mortar. The sample was then incubated at 60°C for 30 min with occasional gentle swirling. The extraction was done once with equal volume of chloroform: isoamyl alcohol in a ratio of 24:1 by mixing gently but thoroughly. The solution was then centrifuged at room temperature at 10000 *g* for 10 min. Aqueous phase of the solution was aspirated with wide bore pipette and transferred to a clean centrifuge tube. Then 2/3^rd^ volumes of cold isopropanol was added and mixed gently and left for 12 h at 4°C to precipitate nucleic acid. It was then spinned at room temperature for 5 min at 10000 *g* and the supernatant was gently poured off as much as possible without losing the precipitate. To the pellet 5 ml wash buffer was added and swirled gently to resuspend DNA. The pellet was washed with wash buffer for 25 min at room temperature. It was followed by a brief spin at room temperature. The supernatant was poured off carefully and the pellet was allowed to air dry briefly at room temperature. Dried DNA was resuspended in 1 ml TE solution.

To check the quality and effect of FA treatment, the extracted DNA was run on 1.2% agarose gel along with molecular weight marker. The gel was run in 0.5X TBE at 100 V.

### Statistical analyses

Experimental design was completely randomized. All the experiments were carried out in triplicates and repeated three times. The mean, standard error and One-way ANOVA were calculated using the average data of the experiment. Analysis of variance (ANOVA) was calculated at 5% probability level according to the method described by Gomez and Gomez ([1984]). The mean seperations were carried out using Duncan’s multiple range tests (Duncan [1955]) and significance was determined at p < 0.05.

## Results

### Fusaric acid induced cell death

**Figure 1:**
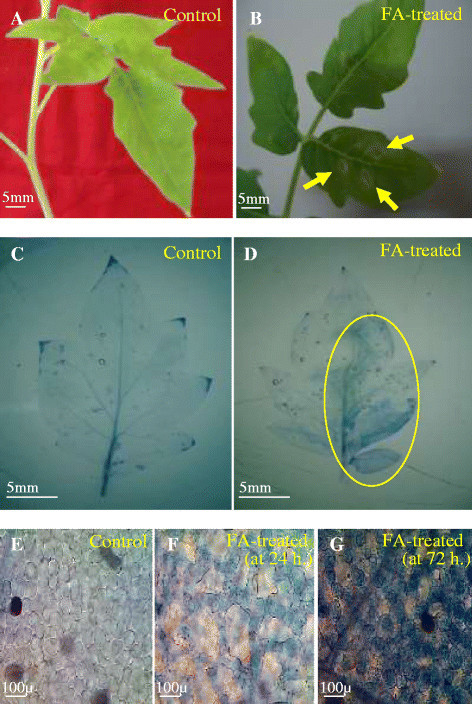
**Effect of FA (250 μg/ml concentration) on the cell death of tomato leaves. (A)** Control leaves, **(B)** FA treated leaves showing necrotic lesions, **(C and D)** Evans blue staining of control and FA-treated leaves showing cell death, **(E)** Microscopic observation of unstained control leaf tissue, **(F and G)** Microscopic observation of stained tissues of FA-treated leaves at 24 h and 72 h after treatment respectively.

**Figure 2:**
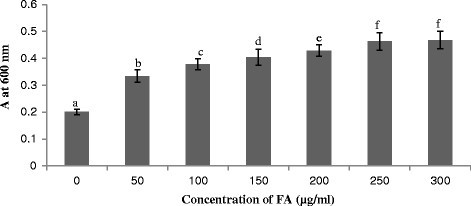
**Cell death in the leaves of tomato plant treated with various concentrations of FA after 72 h (A, Optical density).** Bars represent ± standard error. Different letters indicate that values are significantly different (p < 0.05).

The phytotoxic effect of FA was observed on the leaves of tomato plant. The leaves treated with FA exhibited visible necrotic lesions (Figure 1A, B) as a result of cell death. Evans blue staining was used as a marker of cell death (Figure 1C, D). The dead cells were stained blue whereas the untreated ones appeared unstained under the light microscope (Figure 1E, F, G). The cell death was observed to increase with increasing concentration of FA (Figure 2). Maximum cell death was induced by 250 μg/ml of FA as observed by Evans blue uptake. FA at higher concentration of 300 μg/ml also showed similar amount of cell death and the cell death induced by 250 μg/ml and 300 μg/ml of FA was almost similar. Hence, the concentration of 250 μg/ml for FA was selected for further experiments to induce cell death in the leaves of tomato plant.

### FA induced oxidative burst

**Figure 3:**
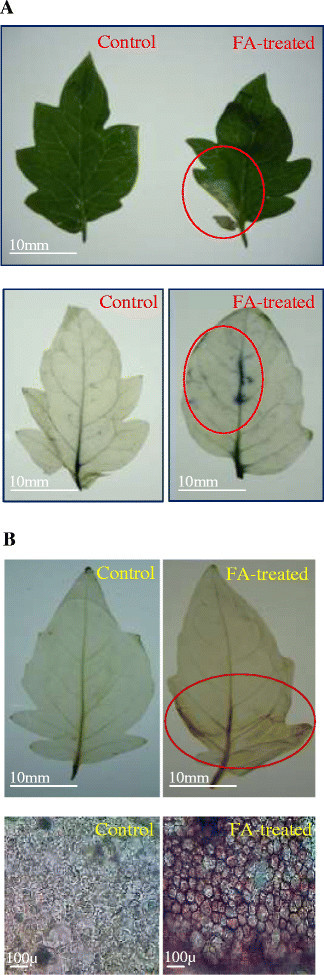
**Detection of ROS generation in the tomato leaves treated with FA. (A)** Accumulation of O_2_^.-^ in the tomato leaves by NBT staining method at 12 h of FA treatment. **(B)** Localization of H_2_O_2_ in the leaves of tomato by DAB staining method at 24 h of FA treatment.

The production of O_2_^.-^ was studied by NBT reduction which gave rise to a purple formazan precipitate (Figure 3A). Intense staining of leaves by NBT confirmed the presence of high amount of superoxide anion radical in the FA treated leaves after 12 h. The accumulation of H_2_O_2_ was visualized by DAB staining where reddish-brown coloration was observed (Figure 3B).

**Figure 4:**
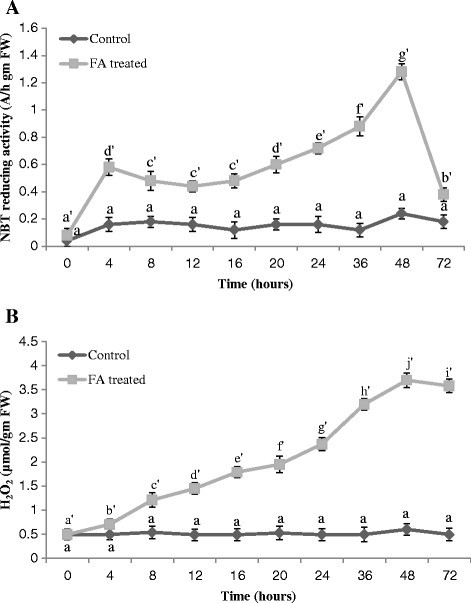
**Measurement of ROS generation in the tomato leaves treated with FA. (A)** O_2_^.-^ production in the leaves of tomato plant treated with FA. Bars represent ± standard error. Different letters indicate that values are significantly different (p < 0.05). **(B)** H_2_O_2_ production in the leaves of tomato plant treated with FA. Bars represent ± standard error. Different letters indicate that values are significantly different (p < 0.05).

In FA-treated leaves, an increase in NBT reducing activity was detected as early as 4 h after the treatment and sustained for 2 days. The result indicated that FA treatment evoked burst of O_2_^.-^ in tomato leaves. The elevated peak was observed at 48 h after the FA treatment, then after the O_2_^.-^ level declined (Figure 4A).

The leaves treated with FA showed enhanced H_2_O_2_ release. The amount of H_2_O_2_ started to increase from 8 h after FA application that reached to maximum at 48 h and decreased thereafter (Figure 4B). The control leaves had a relatively low basal levels of ROS.

### Effect of FA on lipid peroxidation

**Figure 5:**
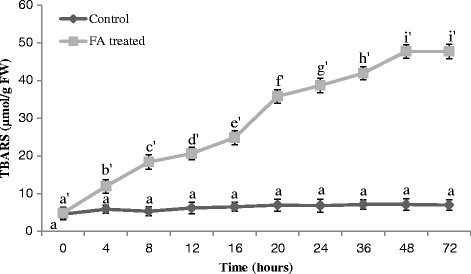
**Lipid peroxidation in the leaves of tomato plant treated with FA.** Bars represent ± standard error. Different letters indicate that values are significantly different (p < 0.05).

The level of MDA, the final decomposition product of lipid peroxidation in the leaves of tomato plants treated with FA was significantly different from control in all sampling. MDA accumulation in leaves was significant after 8 h of treatment and increased gradually and peaked at 48 h, then declined afterwards (Figure 5).

### Ethylene evolution

**Figure 6:**
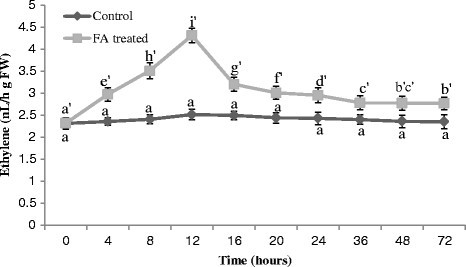
**Ethylene production in the leaves of tomato plant treated with FA. Bars represent ± standard error.** Different letters indicate that values are significantly different (p < 0.05).

A significant increase in ethylene production was observed following the treatment of FA in comparison to untreated tomato leaves. FA triggered ethylene evolution from tomato leaves within 4 h of treatment. With FA treatment ethylene evolution was initially slower but increased gradually with time, reached at peak at 12 h and declined afterwards (Figure 6).

### Activity of antioxidative enzymes

**Figure 7:**
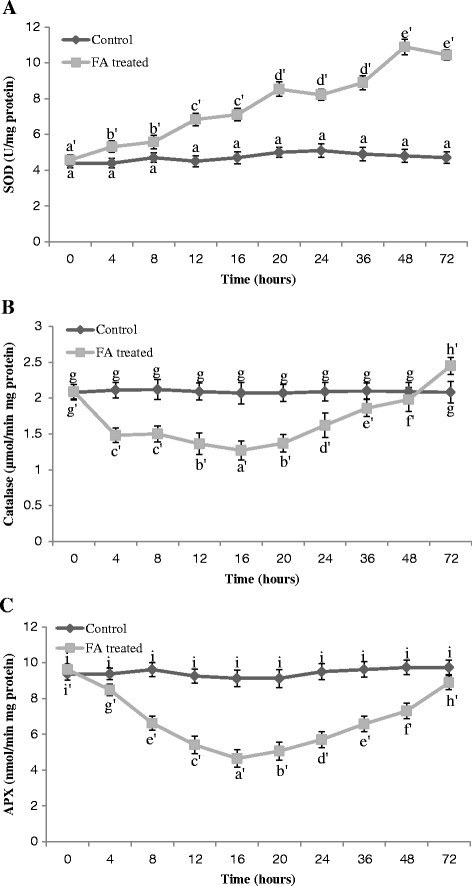
**Activities of antioxidative enzymes in the leaves of tomato plant treated with FA. (A)** Effect of FA treatment on enzymatic activity of SOD in tomato leaves. Bars represent ± standard error. Different letters indicate that values are significantly different (p < 0.05). **(B)** Effect of FA treatment on enzymatic activity of catalase in tomato leaves. Bars represent ± standard error. Different letters indicate that values are significantly different (p < 0.05). **(C)** Effect of FA treatment on enzymatic activity of APX in tomato leaves. Bars represent ± standard error. Different letters indicate that values are significantly different (p < 0.05).

Minimal increase in total SOD activity was observed in the early stages of the experiment, but after 12 h of the FA application an evident increase in SOD activity was observed as compared with the control (Figure 7A).

The H_2_O_2_ scavenging enzymes CAT and APX showed different responses to FA. The activities of CAT and APX decreased before 24 h of FA treatment. After the initial decrease CAT and APX activities increased gradually. Three days after FA application their activities were similar to those as observed in untreated leaves (Figure 7B, C).

### FA-induced DNA degradation

**Figure 8:**
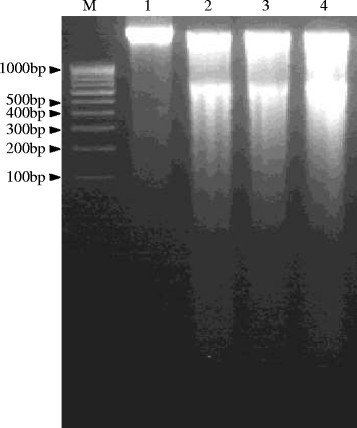
**DNA quality of control as well as FA-treated tomato leaf tissues. Lane M:** Molecular weight marker (100 bp ladder), **Lane 1:** Control leaf tissue, **Lane 2:** FA-treated leaf tissue after 24 h, **Lane 3:** FA-treated leaf tissue after 48 h, **Lane 4:** FA-treated leaf tissue after 72 h.

FA infiltration in the leaves of tomato caused damage to DNA by producing cleavages which was visible in the form of degraded DNA on agarose gel, whereas, the DNA degradation was completely absent in the control leaf tissues (Figure 8).

## Discussion

Research has established that mycotoxin-induced cell death in plants resembles with animal apoptosis (Gilchrist [1997]; Gilchrist [1998]; Krishnamurthy et al. [2000]; Zhang et al. [2009]; Doorn [2011]). Our result demonstrates that FA infiltration/treatment can induce necrotic lesions in treated leaves of tomato. These lesions are reminiscent of the HR lesions (a type of programmed cell death) that are induced by plant-pathogen interactions and other abiotic stress factors. This type of necrotic lesions was also observed when *Arabidopsis* was treated with other mycotoxins such as Fumonisin B1 (Stone et al. [2000]) and AAL-toxin (Gechev et al. [2004]). After infiltration of tomato leaves with FA, symptoms of cell death were observed by Evans blue staining (Figure 1) and also by measuring amount of enhanced ROS levels (Figure 4A, B) as well as by DNA degradation (Figure 8). We estimated the cell death that occurred 24 h after treatment with 250 μg/ml FA as appearance of necrotic lesions on tomato leaves. At 72 h after the incubation period, more number of leaf cells were strongly and positively stained by Evans blue (Figure 1F, G). The dead cells were mostly found in the interior of the lesions.

Reactive oxygen species (ROS) such as H_2_O_2_ and O_2_^.-^ are known toxic metabolic products in plants and other aerobic organisms (Gechev et al. [2006]) and stress factors in plant cells have in common the generation of ROS. The massive, rapid and transient activation of oxidative metabolism immediately after exposure to certain abiotic and biotic stress factors is termed the `Oxidative burst’ in analogy to the `Respiratory burst’, a primary response of mammalian macrophages and neutrophils to invading pathogens (Babior et al. [1997]; Schraudner et al. [1998]; Wohlgemuth et al. [2002]).

In the present study, it was found that O_2_^.-^ generation, measured by means of NBT reduction, was visibly stimulated up to 2 days after FA treatment (Figure 4A). Treatment of FA also enhanced the accumulation of H_2_O_2_. The rate of H_2_O_2_ began to increase later than that of O_2_^.-^ generation and it stayed high between 12 h and 48 h after FA treatment (Figure 4B). Unlike some pathosystems the FA-induced time course of H_2_O_2_ accumulation was not fully coincident with that of O_2_^.-^ generation. Moreover, the timing and intensity of FA-induced O_2_^.-^ and H_2_O_2_ production were distinct from a typical two phase oxidative burst reported for infected plant cells (Baker et al. [1995]; Low and Merida [1996]).

Ethylene is a gaseous phytohormone that has been reported to play an important role in regulating and modulating plant responses including cell death, to both biotic and abiotic stresses (de Jong et al. [2002]). In the present study, ethylene production was highly stimulated by FA treatment (Figure 6) which suggests that ethylene is another major player to bring out the process of cell death in tomato plant. Results demonstrate that ethylene potentiate the oxidative burst, while ROS increases ethylene production. In this way, these signalling molecules stimulate each other’s production, thus amplifying the initial signal, ultimately leading towards plant cell death.

Interestingly, in FA-treated leaves a transient decrease in activity of H_2_O_2_ scavenging enzymes namely CAT and APX was observed (Figure 7B, C). Similar observations were also found in the previous work of Kuzniak et al. ([1999]). The strongest fall in CAT and APX activities was visible within the first day after FA treatment. Similarly, a decline in CAT activity was described in bean leaves following inoculation with *Pseudomonas syringae* pv. *phaseolicola* (Adam et al. [1995]) and lower level of H_2_O_2_ scavenging activity was correlated with increased H_2_O_2_ production during interaction between *Pseudomonas syringae* pv. *glycinea* and soyabean suspension cells (Baker et al. [1995]). The observed decline in CAT and APX activity might have resulted from both an inhibitory effect of FA and an activated down regulation mechanism. The present results suggest that in FA-treated tomato leaves, SOD activity increased and transient decline in both CAT and APX activities may at least partially accounted for the enhanced H_2_O_2_ accumulation.

FA produces oxidative stress which is evident from enhancement in lipid peroxidation (Figure 5). FA may cause molecular damage to plant cells either directly or indirectly through the formation of ROS, which include free radicals as well as non-radical molecules of high reactivity. Membrane lipid especially prone to attack by free radicals (^.^OH, H_2_O_2_), which can convert fatty acids to toxic lipid peroxides, destroying the biological membranes (Foyer et al. [1994]). Since lipid peroxidation is ascribed to oxidative damage (Zenk [1996]), measurement of MDA levels, a common product of lipid peroxidation, is routinely used as sensitive index of oxidative stress (Smirnoff [1993]; Metwally et al. [2005]; Choudhary et al. [2007]). In this study, MDA level rose significantly high when plants were subjected to FA treatment. This suggests that superoxide radicals are produced on FA treatment leading to increased peroxidation.

## Conclusion

Results from experiments are reproducible with respect to the morphological effects and cell death. In our study, a concomitant increase in ROS production, the down regulation of the antioxidant enzymes activities and a change of lipid peroxidation were crucial for the onset of cell death. The present investigations demonstrated that FA-induced lesions on tomato leaves are similar to pathogen-induced lesions (HR) in many respects, such as the occurrence of an oxidative burst, lipid peroxidation, and DNA degradation. FA-induced oxidative burst is evident from enhancement in lipid peroxidation, H_2_O_2_ and O_2_^.-^ levels. However, to cope with the FA toxicity, tomato plants develop a cellular strategy involving activation of various enzymatic antioxidants. This modulation of antioxidative system could reflect a defence response to the cellular damage provoked by FA treatment. The study suggests that the interaction between FA and tomato provides a useful system to study plant cell death.

## Authors’ contributions

In this work, author (VKS) designed and performed experiments, analysed data, and wrote the paper. Another author (RSU) supervised the whole work. Both authors have read and approved the final manuscript.

## Authors’ original submitted files for images

Below are the links to the authors’ original submitted files for images. Authors’ original file for figure 1 Authors’ original file for figure 2 Authors’ original file for figure 3 Authors’ original file for figure 4 Authors’ original file for figure 5 Authors’ original file for figure 6 Authors’ original file for figure 7 Authors’ original file for figure 8

## Abbreviations

APX: Ascorbate peroxidase
ANOVA: Analysis of variance
BHT: Butylated hydroxyl toluene
CAT: Catalase
DAB-HCl: 3,3′-Diaminobenzidine hydrochloric acid
FA: Fusaric acid
FW: Fresh weight
HR: Hypersensitive response
MDA: Malondialdehyde
NBT: Nitroblue tetrazolium
PDB: Potato dextrose broth
PVP: Polyvinyl pyrrolidone
ROS: Reactive oxygen species
SA: Salicylic acid
SDS: Sodium dodecyl sulphate
SOD: Superoxide dismutase
TBA-RS: Thiobarbituric acid-reactive substances
TCA: Trichloro acetic acid
