# Supplementary figures and images for: Fusaric acid induced cell death and changes in oxidative metabolism of Solanum lycopersicum L
Source: Bot Stud. 2014 Aug 27;55:66. doi: 10.1186/s40529-014-0066-2 (PMC5432760; doi:10.1186/s40529-014-0066-2)

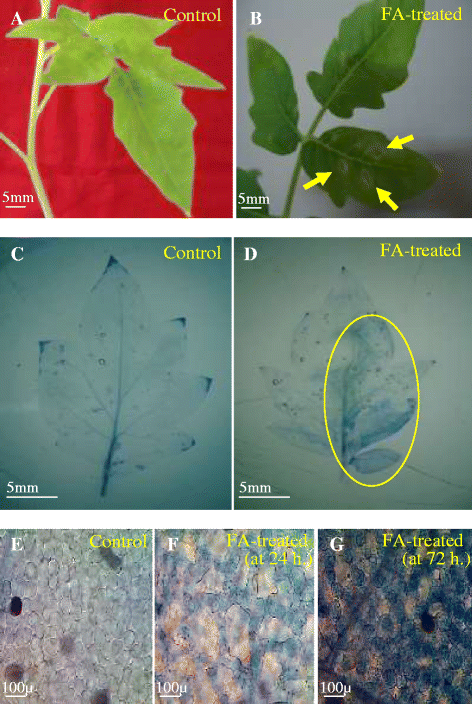

Supplement: Supplementary file 1 — Authors’ original file for figure 1 [file 40529_2014_9066_MOESM1_ESM.gif]

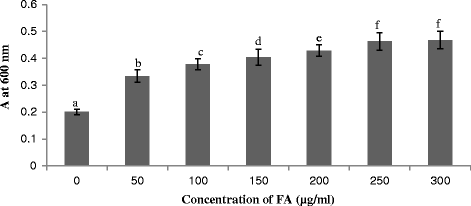

Supplement: Supplementary file 2 — Authors’ original file for figure 2 [file 40529_2014_9066_MOESM2_ESM.gif]

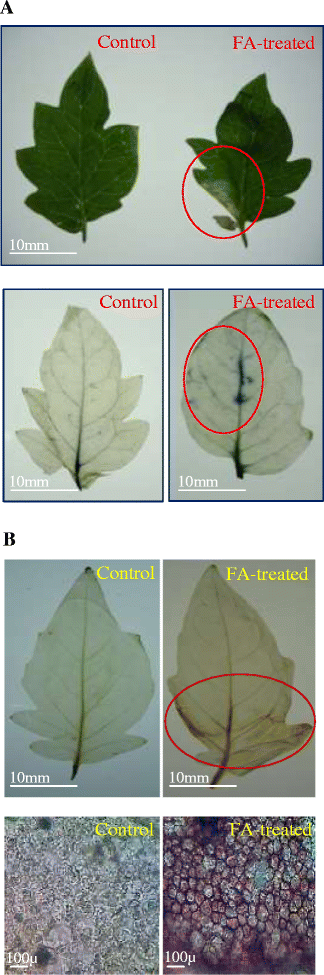

Supplement: Supplementary file 3 — Authors’ original file for figure 3 [file 40529_2014_9066_MOESM3_ESM.gif]

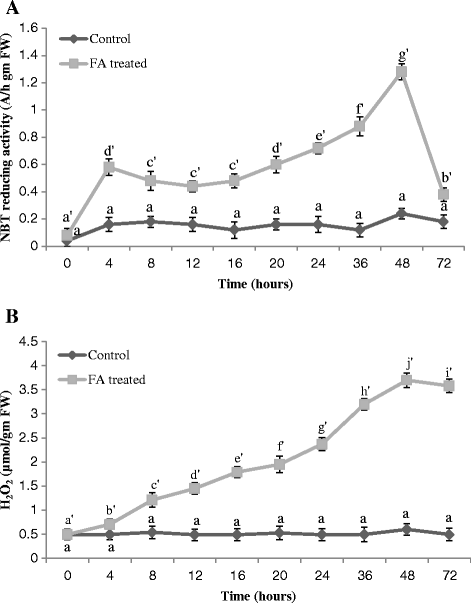

Supplement: Supplementary file 4 — Authors’ original file for figure 4 [file 40529_2014_9066_MOESM4_ESM.gif]

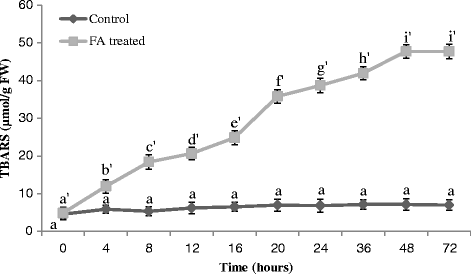

Supplement: Supplementary file 5 — Authors’ original file for figure 5 [file 40529_2014_9066_MOESM5_ESM.gif]

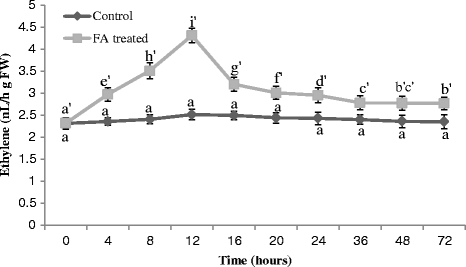

Supplement: Supplementary file 6 — Authors’ original file for figure 6 [file 40529_2014_9066_MOESM6_ESM.gif]

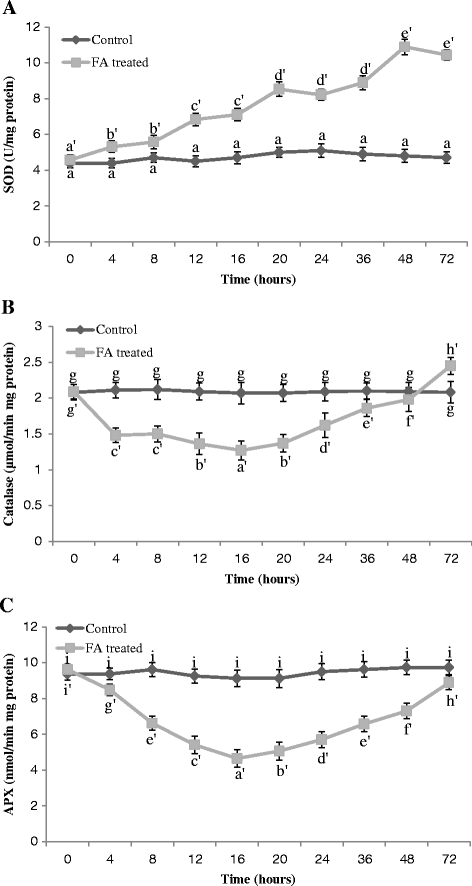

Supplement: Supplementary file 7 — Authors’ original file for figure 7 [file 40529_2014_9066_MOESM7_ESM.gif]

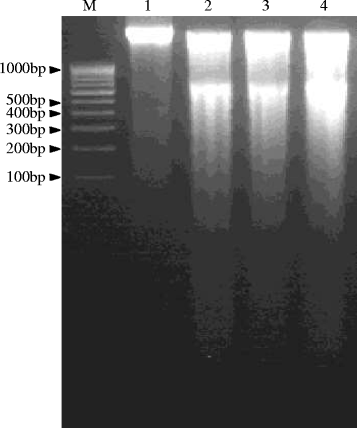

Supplement: Supplementary file 8 — Authors’ original file for figure 8 [file 40529_2014_9066_MOESM8_ESM.gif]
